# Modern Sociology

**Published:** 1900-02-03

**Authors:** 


### Modern Sociology.

### SCHOOLS AND SWIMMING BATHS.

By a Correspondent.

Is it not desirable that swimming should be made a part of our national education? We are a maritime nation; that is our peculiar boast, as it is our best safeguard. Surely, then, our children should be taught to be at home in the water. That of itself might be regarded as being reason enough for adding swimming to the universal curriculum of study; but if it be urged that, after all, comparatively few of our sons become sailors, we must bring forward other pleas. For one, we have every year a number of drowning accidents which might be avoided if every person could swim as naturally as he could walk. Yet that is, perhaps, a personal consideration; and on this ground instruction in swimming should be left to parents as instruction in walking is. The majority of parents in the middle and upper classes may see their responsibility in this matter; but when you come to humbler folks they will fail to perceive the use of spending time and money in giving their children training in what they would regard as an accomplishment, if not as a fad. Thus, the responsibility gets evaded, or is thrust back on the nation again.

Perhaps the question of personal safety would not touch the national conscience, but the question of public health should. As the belief becomes more and more general that many of our diseases are due to dirt, there comes a greater desire to promote universal cleanliness. Now the working classes are not clean. The fact implies no blame to them. How is a man whose work necessitates the soiling of his hands, and often of great part of his person, to keep himself clean when the accommodation for washing provided in his home is limited to a tap of cold water and a small basin, or a sink? We should doubt if in many working-class homes either parents or children get a thorough wash oftener than three or four times a year. There are public baths, indeed, in our large towns, but it costs something to visit them, and the British working man has, or thinks he has, better ways of spending his money than that. And the children, at least when they are too big to be put bodily into the wash-hand-basin, are not likely to be better off in the way of ablution than their parents. Their only chance of getting a thorough wash is to get it at school. And the only way they can get it at school without offending the susceptibilities of their parents is to get it as a lesson in swimming.

There is no doubt that given thus, the children, boys and girls alike, would welcome it. Better even than drill, which all children enjoy, would be the splashing in the water, the fun, the emulation in healthy exercise. A swimming lesson once or twice a week would be looked forward to by almost every child. They would feel better after it, though they would not know that part of this comfortable feeling would spring from being freed from the previous days' accumulation of dirt and perspiration. They would be invigorated, they would be healthier and happier, better fitted for the more formal part of education. With the dirt would be washed off many disease bacilli which some among them were bringing unwittingly to disseminate among their fellows, while in their systems, braced up by the tonic of the bath, they would possess the most efficacious of antiseptics.

Of course, instruction in swimming and the erection of suitable baths implies some addition to the School Board rate, and would therefore arouse some opposition. But even those who do not see that any good can result to the nation from the development of the mental faculties of the children of the poor, might perceive that something was to be gained by making them more healthy. Diseases that are born in the slums have a way of spreading into better quarters. In small places there might be considerable opposition, but in our great cities, where the need for the wholesale washing of the population is greatest, it is doubtful if there would be any marked opposition to the scheme. The cost of providing baths and a bath-master would not be large when spread over a considerable area, and the general spirit of the community would not be unfavourable. And there can be no doubt that the result would be exceedingly healthful.

Moreover, if once a habit of cleanliness were common among the youths who are to be the workers of the next generation, they would be more likely to carry on the custom in maturer years. They would patronise public baths more than they do now; and there would be a strong moral suasion brought to bear upon employers to enable workers to cleanse themselves from the dust and sweat of toil before going home. In all large factories there is steam and power that could be turned on for the purposes of the bath without much trouble or expense. If at present this is wasted, and the worker goes home dirty, let us blame him and not his master. The employer has no reason for thinking that such an addition to the amenities of factory life would be welcome. Indeed, what testimony we have goes to prove the reverse. An American firm (the Palmer Manufacturing Company) provides a basin and towel for each separate one of their thousands of workmen, and expects them to use them before leaving the shop, and to go home looking like gentlemen—as why should they not? But a firm of American origin, which has a factory on this side of the water, introduced and tried to insist on the same thing. Hereupon the spirit of the free-born Briton rose in arms, and there was almost a strike, until the obnoxious regulation was removed. The Briton did not mind going home dirty, and making his good-wife a slattern through sheer hopelessness at the difficulty of keeping the house clean when her husband came home with clothes and person dirty. If he did object to going through the streets in a condition that makes fastidious people draw away from him, not through pride but through a love for cleanliness, if he felt any real desire to be clean, he would get what he wants; for in this country everyone can get what he wants provided only that he can get a sufficient number of his neighbours to share in the desire. Wherefore by all means let us try to make our fellow-citizens desire to be clean. They will profit by it, and so shall we all. And the best way to make them cultivate this virtue is to teach it to them in their youth by means of compulsory cleanliness in their school days. The children would be willing, and in after-days might be grateful. And the increase of cleanliness on the part of the children of the humbler classes would not be without benefit to the community at large.

